# 

**Published:** 1851-01

**Authors:** 


### INDEX.

A good hit at homœopathy 133
Abdomen, wound of 489
Abscess of cancellated structure of the tibia, 33
Absorption of aliments 276
Acetate of lead in epistaxis 765
Acetate of potash in some diseases of skin 70
Account of a man who lives on large quantities of raw flesh 433
Aconite, accidental poisoning by 680
Aconite in treatment of lichen, 683
Acupuncture in treatment of unconsolidated fracture of thigh, 735
Address on Medical Jurisprudence. By H. N. Storer, M.D., &c., bib. notice of 572
Administration of cod-liver oil 552
Adulterated drugs in New York 200
Agave Americana as a remedy for scorbutus 741
Ague, treatment of, in Morocco 609
Air in the heart 270
Alcoholic liquors, use and abuse of, in health and disease. Prize Essay. By Wm. B. Carpenter, M. D., &c., bib. notice of 260
Alleged mal-practice 593
Aloes, cathartic principle of 620
Alome 620
Alum treatment of lead colic 201
Amaurosis in albuminuria 679
American antidote against snake poison 139
American Medical Association 130, 400, 410
American Medical Association, transactions of 57
Amputation of the leg 29
Amputation of half the lower jaw 30
Amputation of the mammary gland 33
Amussah on use of water in surgery 573
An act for registration of births 336
Anæsthetic agents in ancient China 337
Anæsthetic, new local 362
Anasarca in disease of the heart 678
Anatomy, Surgical. By J. Maclise, Surgeon, bib. notice of 532, 722
Anatomy, general and pathological elements of. By D. Craigie, M. D., &c., bib. notice of 578
Anatomy, physiology and pathology of the eye. By H. Howard, M. R. C., L. S., &c., bib. notice of 259
Anatomy of the urethra 477
Anatomical preparations, preservation of, by arseniuretted hydrogen gas 344
Aneurism of common carotid artery 544
Animal magnetism, letters to a candid inquirer on. By W. Gregory, M. D., &c., bib. notice of 574
Ankylosis, Mütter's lectures on 37
Anthelmintic virtues of kousso 341
Antiseptic properties of chloroform 273
Antiperiodics 69
Anus, imperforate 479
Apparatus for transfusion 673
Apparatus for treatment of fractures of inferior extremities 637
Appointment of Dr. Thomas Stewardson 461
Appointment of Dr. A. Smith 267
Appointment of Dr. E. Bartlett 417
Appointment of Dr. A. C. Post 417
Appointment of Dr. M. Clymer 417
Appointment of Dr. Pereira 802
Appointments, hospital 337
Army, rank of medical officers in 328
Arsenic, sale of 338
Arsenious acid, poisoning by 153
Artificial limbs 34
Articular rheumatism, treated with lemon juice 202, 680
Assistant Surgeons, U. S. Navy 200
Astragalus, excision of 343
Bell's grave 596
Bernard on absorption of aliments 276
Bibliographical notices 57, 112, 182, 252, 307, 369, 439, 517, 569, 657, 722
Bladder, extraction of foreign bodies from 616
Blind gut of fowls as a plug in epistaxis 614
Blood, casein in; synthetical proof by the formation of artificial milk 473
Blood, transfusion of 670
Bone, resection of 644
Bones, new method of whitening 766
Bougon, Dr. death of 463
Bowels, treatment of obstructions of the 804

British provincial, medical and surgical journal 669
Bromide of iron, remarks on the use of 758
Bronchocele, extirpation of 738
Bronchotomy in œdmatous laryngeal angina 683
Buffalo Medical College 462
Burns, collodion applied to 205
Calcis, fracture of anterior portion of 286
Calculus removed by dilatation of the urethra, 275
California, remarks on 21, 91
Carbonate of soda, sudden death from long continued use of 71
Card, Dr. Ramsay's 800
Card, Dr. Robertson's 801
Carotid artery, aneurism of 544
Casein in the blood 473
Case of severe wound by a circular saw 774
Cases from Dispensary practice of Pennsylvania College 182
Case of hydrophobia in South Easton 221
Cases of plastic surgery 238
Case of gun shot wound 17
Calomel in large doses as a diuretic in dropsies 770
Catalogues of students 200
Cazeaux, election of 463
Chart of the signs furnished by auscultation and percussion, and of their application to the diagnosis of diseases of the lungs and heart. Revised and arranged by P. C. Gouch, A. M., M. D., &c., bib. notice of 64
Charge to graduates of Jefferson Medical College. By T. D. Mütter, M. D., bib. notice of 373
Charge to Graduates of Pennsylvania College. By W. Darrach, M. D., bib. notice of 373
Chair of medicine in faculty of Paris 592
Chemistry, review of for students. By J. G. Murphy, M. D., bib. notice of 190
Chemistry of respiration 478
Child, case of malformation in 429
Children, cases of obscure pneumonia in 1
Chinoidine in intermittent fever 231
Chloroform, antiseptic properties of 273
" fatal effects of 341
" in the treatment of poisoning by strychnia 542
Chloroform, deaths from 681
Chloroform, solution of gutta percha in 682
Cholera 224
" tannin in 679
Chorea, case of, treated successfully with carbonate of iron 471
Chronic vomiting as symptomatic of disease of the kidneys 740
Ciliary movements in the human subject 755
Clairvoyance in the 17th century 671
Clinical reports 28, 95, 164, 253, 300, 501
Clinic of Jefferson Medical college, 35, 102, 164, 245
Clinic of Penna. Hospital 109, 300
Codex, French 133
Cod liver oil 467
" administration of 552
Coffinism 730
Collodion applied to burns 205
" in skin diseases 543
" application of, in occlusion of the eyelids 738
Colleges, classes and graduates, list of, for 1851 336
Colon, obstruction of 686
Complimentary to the profession 726
Compression of the aorta in uterine hemorrhage 746
Concours 466
Conjunctiva, nitrate of silver stains of 740
Constricted vagina 696
Coroners, medical 338
Corpulence, enormous 594
Corrections 376
Correspondents, notice to 533
Cutaneous galvanism 670
Cyanide of potassium, poisoning with 617
Cyanide of silver, poisoning with 617
Cystic goitre 501
Deaths from chloroform 681
Death of Dr. J. F. Fitts 726
" Wheaton 729
" Rayer 729
" H. K. Burroughs 729
" A. F. Stone 729
" Oken 729
" Kidd 729
" Baudelocque 739
" S. G. Morton 382
" S. Sergeant 461
" Bougon 463
" M. H. Smith 464
" G. Lillington 588
" J. M. Wallace, 799

Death of Dr. Stephen Harris 799
" G. Sharp Pattison 799
" J. Kearney Rogers 799
" J. R. Manly 799
" Frederick Crowley 796
" Dr. John Baskin 797
" Roe 803
" De Savigny 803
" Mr. S. Graham 729
Deaths of distinguished physicians 133, 199, 269
Deformed fracture, resection of femur for 32
Delegates to American Medical Association 199
Delegates from Philada. county Soc. 269
Delegates to the National Medical Convention from Chester county 802
Deligation of common carotid for aneurism 544
Demonstrative medicine 50
Demonstrative surgery in University of Pennsylvania 28, 95, 174, 233
Dengue 339
Dental medicine, a practical treatise on, being a compendium of medical science, as connected with the study of dental surgery. By T. E. Bond, A. M., M. D., &c., bib. notice of 186
Deposits, urinary, their diagnosis, pathology, and therapeutical indications. By G. Bird, M. D. &c. bib. notice of 533
Despotism in Prussia 673
Diameters of the fœtal head 634
Diarrhœa, a hitherto unnoticed symptom of menstruation 481
Dictionary of medical science, containing a concise explanation of the various subjects and terms of physiology, pathology, hygiene, &c. &c. By R. Dunglison, M. D., &c., bib. notice of 722
Dilatation of urethra, for removal of calculus 275
Digitaline 742
Disease of kidneys, chronic vomiting in 746
Disease of heart, anasarca in 678
Disease of the potato 672
Disease of pancreas 472
Discouragement of vicious advertisements 337
Diseases and surgical operations of the mouth and parts adjacent, treatise on, by M. Jourdain, dentist, &c., bib. notice of 122
Diseases of menstruation and ovarian inflammation, in connection with sterility, pelvic tumours, and affections of the womb. By E. J. Tilt, M. D., &c., bib. notice of 311
Dislocated elbow, reduction of, after six weeks 30
Dislocation of the lens 34
" scaphoid bone of the foot 203
Dislocation of humerus on dorsum of scapula 614
Dispensatory of U. S. of America, By G. B. Wood, M. D., and F. Bache, M. D., bib. notice of 667
Distorted knee, examination of 612
Distribution of blood vessels in the mucous membrane of the stomach 755
Drugs adulterated in New York 200
Dulcamara, poisoning with 71
Dura mater, fungus of 34
Early struggles of Leuret 462
Early viability 676
Edematous angina, bronchotomy in 683
Editorial 66, 176, 191, 265, 328, 460, 576, 533, 796
Education, medical 57
Effusion behind the uterus 551
Election of M. Nelaton 466
Election of M. Cazeaux 463
Electro-magnetism in the treatment of poisoning by opium 357
Elements of general and pathological anatomy, &c. By D. Craigie, M. D., &c., bib. notice of 578
Elements of physiology, including physiological anatomy. By Wm. B. Carpenter, M. D., bib. notice of 724
Emigrants' Hospital 466
Encysted tumours, treated with injections of iodine 205
Enormous corpulence 594
Epistaxis, acetate of lead in 765
Epistaxis, use of the blind gut of fowls in 614
Epilepsy, treated by tracheotomy 617
Eruptive fevers, lectures on. By G. Gregory, M. D., &c., bib. notice of 453
Ether and chloroform, their employment in surgery, dentistry, midwifery, &c. By J. F. B. Flagg, M. D. &c., bib. notice of 264
Examination of a distorted knee 612
Excision of joints 343

Excision of the astragalus and os calcis 343
Experiments on the pancreatic fluid of the calf 478
Experiments on the effects of nicotine 682
Explanations 376, 752
Exostosis of nasal bones 33
External poisoning, tr. of iodine in treatment of 479
Extirpation of goitre 205
Extirpation of left testicle 33
Extirpation of a voluminous bronchocele 738
Extraction of foreign bodies from the bladder 616
Extraordinary accident 672
Facial paralysis 679
Family and ship medicine chest companion; being a compendium of domestic medicine and surgery, and materia medica, with directions for the diet, and management of the sick room, &c. By a practising physician, bib. notice of 65
Fatal effects of chloroform 341
Fearnside on disease of the pancreas 472
Femur, resection of, for deformed fracture 32
Fever, scarlet, lectures on 73, 141, 209, 277, 345, 417, 553, 621, 689
First principles of medicine. By A. Billing, A. M., M. D., &c., bib. notice of 189
Fiske fund prize 592
Fistula of stomach 199
" urethra 613
" vesico-vaginal 650
Fœtal head, diameters of 634
Foot prints, means of solidifying 484
Foot, new operation on the 540
Fracture of neck of humerus 616
" anterior portion of os calcis 286
Fractures of inferior extremity, apparatus for treatment of 637
Friction with chloroform in treatment of traumatic tetanus 737
French codex 133
Galvanic poultice 341
Galvanism, cutaneous and mnscular 670
General and comparativephysiology, principles of. By W. B. Carpenter, F. R. S., &c., bib. notice of 517
Generation, spontaneous 275
Geological observer. By Sir H. T. De La Beche, C. B. &c., bib. notice of 668
Gillespie on bromide of iron 158
Gland, parotid, removal of 30
Goitre, cystic 501
" extirpation of 205
Gonorrhœa, hydrastis canadensis in treatment of 733
Gorini, Professor 803
Graduates in medicine 267
Gunshot wound, case of 17
Happiness in its relations to work and knowledge. By J. Forbes, M. D., &c., bib. notice of 579
Hare-lip, operation for 686
Heart, air in the 270
" theory and formation of valvular diseases of 748
Hernia, obturator, relieved by an operation 542
Hernia of the ovary mistaken for encysted tumor 744
Hints on the every day duties of the surgeon. By H. H. Smith, M. D., bib. notice of 372
Historical sketch of surgery. By J. Bryan, M. D., bib. notice of 669
History of medical education and institutions in the U. S., &c. By N. S. Davis, M. D., &c., bib. notice of 315
Homœopathy, a good hit at 133
Honorary distinctions offered to M. Ricord 59
Honors to medical men in England 132
Hospital for sick children in London 337
Hospital appointments 337
Hotel Dieu, staff of 592
Hot springs on the overland route to California 775
Humerus, dislocation of, on the dorsum of the scapula 614
Hunterian museum 594
Hydrastis canadensis in the treatment of gonorrhœa 733
Hydrophobia, case of in South Easton 221
Hyperacusia in facial paralysis 679
Illness of assistant physicians at Wards Island Hospital 466
Illustrations of syphilitic disease. By P. Ricord, D. M. P., &c., bib. notice of 439
Imperforate anus 479
Iudian hemp as an excitant of uterine contractions 677
Indolent ulcer caused by electric moxa 481
Inhalation, mercurial 368

Inhalation of chloroform, new application of 688
Influence of the hours of the day on mortality 606
Intermarriage, or the mode in which, and the causes why, beauty health and intellect result from certain unions, and deformity, disease and insanity from others. By A. Walker, bib. notice of 371
Intermittent fever, chinoidine in 231
Introductory lecture on happiness in its relations to work and knowledge. [illegible]y J. Forbes, M. D. bib. notice of 579
Introductory to the course of lectures on the theory and practice of medicine, in the University of Pennsylvania. By G. B. Wood, M. D., bib. notice of 124
Introductory lecture on the impediments to the study of medicine. By J. K. Mitchell, M. D., bib. notice of 124
Introductory lecture to the course of materia medica and pharmacy. By J. Carson, M. D., bib. notice of 124
Introductory lecture, delivered at the Medical College of Ohio, Nov. 4th, 1850. By J. Bell, M. D., bib. notice of 124
Introductory lecture to the course of institutes, by Samuel Jackson, M. D., bib. notice of 124
Introductory address to the class of Pennsylvania College. By W. L. Atlee, M. D., bib. notice of 124
Introductory address delivered at the St. Louis University. By M. M. Pallen, M. D., bib. notice of 124
Inversion of vagina, coming on during labor 745
Iodine 467
Iodine injections in the treatment of encysted tumours 205
Iris, prolapsus of 88
Iron, bromide of, remarks on the use of 158
Jenner, monument to 588
Kentucky State Medical Society 802
Kiestine 745
Kirkbride's report of the Pennsylvania Hospital for the insane, for the year 1850, bib. notice of 324
Kousso 467
" anthelmintic virtues of 341
Lactation 206
Lancet, Philadelphia 131
Lassaigne on the pancreatic fluid of the calf 478
Laws of health in relation to mind and body. By L. J. Beale, M.D., &c., bib. notice of 666
Lead colic, alum in treatment of 201
Lead palsy resulting from the use of soda water contaminated with lead 807
Lecture on ankylosis 37
Lectures on scarlet fever 73, 141, 209, 277, 345, 417, 553, 621, 689
Lectureship on dental surgery in medical colleges 295
Lectures on the eruptive fevers. By G. Gregory, M.D. &c.; bib. notice of 453
Lectures on digestion, respiration and secretion 597
Left testicle, extirpation of 33
Leg, amputation of 29
Lemon juice in acute articular rheumatism 202, 679, 680, 806
Lens, dislocation of 34
Leuret, early struggles of 462
Letters to a candid inquirer on animal magnetism. By W. Gregory, M.D.; bib. notice of 574
Lichen, use of aconite in 683
Life insurance offices, medical certificates for 198
Ligature, subcutaneous, application of, for deep-seated nævi 274
Limbs, artificial 34
List of colleges, classes and graduates, for 1851 336
London Medical Examiner 726
London, Sydenham Society of 131, 533
Lord Langdale 463
Lower jaw, amputation of 30
Luxation of penis 204
Lying-in hospital of the Parisian faculty 669
Maguey, as a remedy for scorbutus 741
Malformation in a child, case of 429
Malpractice, prosecution for 728
Mammary gland, extirpation of 33
Materia medica 129, 273
M'Munns's Elixir, substitute for 618
Means of solidifying foot-prints 484
Medicine, demonstrative 50
Medical education, report on 57
Medicine, first principles of. By A. Billing, A.M., M.D., &c.; bib. notice of 189

Medical students' guide in extracting teeth; with numerous cases in the surgical part of dentistry; with illustrations. By S. S. Hornor, practical dentist; bib. notice of 130
Medical officers in the navy, rank of 191
Medical certificates for life insurance offices 198
Medical news 199, 267, 417, 461, 586, 669, 726, 802
Medical education and institutions of the United States, &c. By N. S. Davis, M.D., &c.; bib. notice of 315
Medical coroners 338
Medical Commencement of the University, with valedictory address. By W. E. Horner, M.D.; bib. notice of 373
Medical prescriptions 464
Medical properties and uses of the Cimicifuga racemosa 807
Medical department of St. Louis University 464
Medical wards of St. Joseph's Hospital 498
Medical Society of London 540
Medical jurisprudence, an address on. By D. H. Storer, M.D., &c., bib. notice of 572
Medical appointments 592
Medical reports, Southern. By E. D. Fenner, M.D.; bib. notice of 665
Medical officers of the Parisian hospitals 672
Medical department of U. S. Army 725
Medical schools of Philadelphia 726
Medical Examiner, London 726
Medical periodicals in Egypt 731
Medical officers in Roman army 732
Medical properties of skull-cap 741
Medicinal inhalations 705
Medulla oblongata, tumor compressing right side of 753
Meeting of Medical Association of West Penn. and Eastern Ohio 588
Meningitis, tubercular 485
Menstruation, use of purgatives in 481
Mercurials in treatment of typhoid fever 137
Mercury by inhalation 368
Mesmerism 462
Microscopical observations on tumors 718, 757
Microscopic observations respecting arterial and capillary circulation 767
Microscopist, or a complete manual on the use of the microscope, &c. By J. H. Wythes, M.D., bib. notice of 583
Midwifery, theory and practice of. By F. Churchhill, M.D. &c., bib. notice of 373
Minor Surgery, or hints on the every day duties of the surgeon. By H. H. Smith, M.D., &c., bib. notice of 372
Minutes of the proceedings of the Medical Society of Georgia, bib. notice of 585
Mind and body, laws of health in relation to. By L. J. Beale, M.D. &c., bib. notice of 666
Miss Martineau 267
Mistake, corrected 166
Monument to Jenner 588
Mucous membrane of the appendix vermiformis cæci and colon, structure of 85
Munificent legacy 731
Muscular galvanism 670
Museum, Hunterian 594
Mütter's lecture on ankylosis 37
Nasal bones, exostosis of 33
Nashville Journal of medicine and surgery 385, 592
National Medical Association 586
Navy, rank of medical officers in 191
Neck of humerus, fracture of 616
Nelaton, M., election of 466
Neuralgia simulated from swallowing a pin 685
New anti-periodics 69
New hospital 335
New Jersey Medical Reporter 337
New local anæsthetic 382
New method of whitening bones 766
New Orleans Monthly Medical Register 796
New remedies, with formulæ for their administration. By R. Dunglison, M.D. &c., bib. notice of 369
New York medical college 462
New mode of taking fees 463
New mode of administering cod-liver oil 552
New operation on the foot 540
New Hampshire Medical Society 591
New sign language for deaf mutes. By A. J. Meyer, bib. notice of 668
New York Medical Times 725
Nicotine, experiments on the effects of 682

Nicotine, poisoning with 575
Nitrate of silver in leucorrhea 811
Notice to Correspondents 533
Obituary 338, 673
Obscure pneumonia in children 1
Observations on excision of joints 343
" on nitrate of silver stains of the conjunctiva 740
Observer, Geological. By Sir H. T. De La Beche, C.B. &c., bib. notice of 668
Obstetrics 206
Obstetrician's toilet 677
Obstruction of the colon 686
Obturator hernia relieved by an operation 542
Occlusion of the os uteri 696
" of the eyelids, by the application of collodion 738
Oil of the male fern in expulsion of tape-worm 203
Operation for hare-lip 686
Operative surgery. By F. C. Skey, F.R.S., &c., bib. notice of 316
Operative surgery, a system of, based upon the practice of surgeons in the United States, &c. By H. H. Smith, M.D., &c., bib. notice of 724, 781
Operative Surgery based on normal and pathological anatomy. By J. F. Malgaigne, Professor Agrege, &c., bib. notice of 781
Ophthalmic diseases, tannin in 542
Opium, poisoning by 349
Organization of the Profession in Kentucky 669
Original communications 1, 73, 141, 209, 277, 345, 417, 485, 553, 621, 689, 759
Os calcis, fracture of 286
" excision of 343
Outlines of chemistry for the use of Students. By William Gregory, M.D., bib. notice of 794
Ovarian inflammation and diseases of menstruation, in connection with sterility, pelvic tumors and affections of the womb. By E. E. J. Tilt, M.D., &c., bib. notice of 311
Ovariotomy, case of 688
Pancreas, disease of 472
" schirrus of 640
Pancreatic fluid of the calf, experiments on 478
Panum on casein in the blood 473
Parotid gland, removal of 30
Paronychia 505
Pathology and practice of medicine 134, 201, 339, 467
Pathology of varioceles 734
Penis, luxation of 204
Pennsylvania Hospital 109, 777
" " clinic of 300
Pennsylvania State Medical Society 389
Perforation of intestinal canal by worms 678
Petrified bean in the urethra 343
Pharmacopœia of United States, by authority of National Medical Convention, &c., bib. notice of 307
Philadelphia Lancet 131
Philadelphia Association for medical instruction 267
Physicians' Visiting List, diary and book of engagements for 1852, bib. notice of 723
Physiological effects of ascending to great heights 76
Physiology, elements of, including physiological anatomy. By W. B. Carpenter, M.D., &c., bib. notice of 724
Physiology, anatomy and pathology of the eye. By H. Howard, M.R.C., &c., bib. notice of 259
Phosphate of iron and quinia, solution of 552
Plastic surgery, cases of 238
Pneumonia in children 1
Poisoning from opium successfully treated by electro-magnetism 357
Poisoning by strychnia, case of 552
" arsenious acid 153
" aconite 680
" mistake 268
" cyanide of potassium 617
" " silver 617
" dulcamara 171
" secale cornutum 70
" sulphate of iron 617
" nicotine 595
Poultice, galvanic 341
Post mortem duration of the ciliary movements in the human subject 755
Potato disease 672
Potash, acetate of, in diseases of the skin 70
Practical treatise on dental medicine, being a compendium of medical science, as connected with the study of dental surgery. By Thos. E. Bond, A.M., M.D., &c. bib. notice of 186
Prescriptions, medical 464
Prize of the Fiske fund 592

Practical treatise on the diseases and injuries of the urinary bladder, the prostate gland and urethra. By S. D. Gross, M.D., &c., bib. notice of 657
Preservation of anatomical preparations by arseniuretted hydrogen gas 344
Principles of physiology, general and comparative. By W. B. Carpenter, F.R.S., &c., bib. notice of 517
Probe, St. Louis 131
Proceedings of Medical Association of Alabama, bib. notice of 327
Professor Jackson on the Spirometer 51
Professor Kolliker 464
Professor of Chemistry in the Harvard University 200
Prolapsus iridis, case of 88
Prophylaxis of syphilis 737
Proportion of physicians to the population of Virginia 200
Proposition to establish a lectureship on dental surgery in medical colleges 295, 362
Prosecution for malpractice 728
Prurigo, aconite in 683
Prussia, despotism in 673
Purgatives, use of, in different epochs of menstruation 481
Quarantine Congress at Paris 802
Quarterly Summary of the transactions of the College of Physicians, bib. notice of 372, 569
Quinia and iron, solution of the phosphate of 552
Rank of medical officers in the U.S. army 328
Rank of medical officers in the U.S. navy 191
Readers, to our 66
Record of medical science 69, 134, 201, 270, 339, 471, 597, 674, 804
Registration of births 265
Remarks on California 21, 91
Removal of parotid gland 30
Remarks on use of bromide of iron 158
Remedies, new, with formulæ for their administration. By R. Dunglison, M.D., &c., bib. notice of 369
Reports, clinical 28, 95
Reports on medical literature, hygiene, adulterated medicines, medical botany and surgery, bib. notice of 112
Report of Committee on statistics of calculous disease in Ohio. By E. H. Davis, M.D., bib. notice of 370
Report on medical education 57
Report of a general plan for the promotion of public and personal health, devised, prepared and recommended by the Commissioners appointed under a resolve of the Legislature of Massachusetts, &c. &c., bib. notice of 252
Report of Penn. Hospital for Insane for the year 1850. By T. S. Kirkbride, M.D., &c., bib. notice of 324
Report on the climate at Aiken, S.C. 360
Reporter, New Jersey Medical 337
Representation in the American Medical Association 431
Resection of bones 644
Resection of femur for deformed fracture 32
Resignation of Dr. E. Bartlett 335
" T. Reyburn 335
" S. D. Gross 417
Respiration, chemistry of 478
Results of surgical operations in malignant diseases 536
Reunion of wounds of the spinal cord 754
Review of chemistry for students. By John G. Murphy, M.D., bib. notice of 190
Rheumatism, termination of, in suppuration 201
Rich Surgeon 417
Ricord's illustrations of venereal disease 131
Rigidity of cervix and os uteri in cases of parturition, use of tartar emetic in 270
Rupture of uterus, case of 291
Sale of arsenic 338
Sanguineous effusion behind the uterus 551
Scabies, treatment of 739
Scaphoid bone of foot, dislocation of 253
Scarlet fever, lectures on 73, 141, 209, 277, 345, 417, 533, 621, 689
Scirrhus of the pancreas 640
Skull cap, medical properties of 741
Scutellaria laterifolia 741
Secale cornutum, poisoning with 70
Secondary hemorrhage 674
Sedative plan of treatment in cases of fever and disturbed nervous functions, after delivery 547
Sick children, hospital for in London 337
Sign language for deaf mutes. By A. S. Meyer, bib. notice of 668

Simulated neuralgia from swallowing a pin 685
Small-pox, fatal case of, occurring 3d time after vaccination 479
Small-pox, treatment of, in Morocco 609
Smith, Dr. Andrew, appointment of 267
Smith, H., death of 464
Snake poison, antidote against 139
Solution of gutta percha in chloroform 682
Solution of phosphate of iron and quinia 552
Southern medical reports. By E. D. Fenner, M.D., bib. notice of 665
Special anatomy and histology. By W. E. Horner, M.D., bib. notice of 665
Spectacles, their uses and abuses in long and short-sightedness. By J. Sichel, M.D., &c., bib. notice of 524
Spinal cord, reunion of wounds of 754
Spirometer, Prof. Jackson on 51
Spontaneous generation 275
Skin diseases, collodion in 543
" treatment of in Morocco 609
St. Louis Probe 131
Staff of Hotel Dieu 592
Staphyloma 34
Statistical report on diseases of the heart 610
Statistics of calculous disease in Ohio, report of Committee on. By E. H. Davis, M.D., bib. notice of 370
Stethometer 134
Stethoscope and Virg. Med. Gazette 131
Stomach, fistula of 199
" wound of 162
Stout on hydrophobia 221
Strabismus, convergens and divergens 509
Structure of the mucous membrane of the appendix vermiformis cæci and colon 85
Strychnia, chloroform in case of poisoning by 552
Students, catalogues of 200
Study of anatomy in Egypt 732
Sudden death from the long-continued use of carbonate of soda 71
Sudden death after delivery 270
Subcutaneous application of ligature for deep-seated nævi 274
Successful case of ovariotomy 688
Sulphate of iron, poisoning with 617
Summary of demonstrative surgery 28, 95, 174, 233
Surgery 203
" historical sketch of. By J. Bryan, M.D., bib. notice of 669
Surgery, operative. By F. C. Skey, F.R.S., &c., bib. notice of 316
Surgical anatomy. By J. Maclise, Surgeon, bib. notice of 332, 722
Surgical operations in malignant diseases, results of 536
Surgical wards of St. Joseph's Hospital 499
Sweating sickness 594
" report on 751
Sydenham Society of London 131, 533
Syphilis, treatment of, in Morocco 609
" prophylaxis of 737
Syphilitic disease, illustrations of. By P. Ricord, D.M. P., &c., bib. notice of 439
System of operative surgery, based on the practice of surgeons in the United States. By H. H. Smith, M.D., bib. notice of 724, 781
Tannin in cholera 679
" ophthalmic diseases 542
Tape-worm, expulsion of with the oil of male fern 203
Tartar emetic in treatment of rigidity of the os and cervix uteri 270
Tennessee hospital for the insane 726
Termination of acute rheumatism in suppuration 201
Testicle, extirpation of 33
Testimonial to Dr. Laycock 595
Tibia, abscess in the cancellated structure of 33
Theory of the formation of valvular diseases of the heart 748
Theory and practice of midwifery. By F. Churchhill, M.D., &c., bib. notice of 373
Therapeutics 139, 273
Thermal ventilation and other sanitary improvements, applicable to public buildings, &c. By Jno. Watson, M.D., &c., bib. notice of 326
Thermometrical report on the climate at Aiken, S. C. 360
Thyroidal hernia relieved by an operation 542
Tincture of iodine in the treatment of external poisoning 479
Tobacco, use of 67
Tracheotomy in the treatment of epilepsy 617

Toilet of the obstetrician 677
Transactions of the American Medical Association 57, 112
Transfusion of blood 607
" apparatus for 673
Traumatic tetanus, treated by friction with chloroform 737
Treatise on the diseases and injuries of the urinary bladder, prostate gland and urethra. By S. D. Gross, M.D., &c., bib. notice of 657
Treatise on the diseases and surgical operations of the mouth and parts adjacent, with Notes, &c. By M. Jourdain, Dentist, &c., bib. notice of 122
Treatment of lead colic by alum 201
" scabies 739
" skin diseases, syphilis, small-pox and ague in Morocco 609
Treatment of typhoid fever by mercurials 137
Treatment of varicocele 734
Trial for alleged malpractice 573
Tubercular meningitis 485
Tumor compressing the right side of the medulla oblongata 753
Tumors, microscopic observations on 718, 787
Ulcer, indolent, cured by electric moxa 481
Unconsolidated fracture of the thigh treated by acupuncture 735
University of Edinburgh 669
Urethra, anatomy of 477
" petrified bean in 343
" fistula of 613
Urinary deposits, their diagnosis, pathology and therapeutical indications. By G. Bird, M.D., &c., bib. notice of 533
Use of tobacco 67
Use and abuse of alcoholic liquors in health and disease. By W. B. Carpenter, M.D., &c., bib. notice of 260
Use of anæsthetic agents in ancient China 337
Uses and abuses of spectacles in long and shortsightedness. By J. Sichel, M.D., &c., bib. notice of 524
Uterine contractions induced by Indian hemp 677
Uterine hemorrhage, compression of the aorta in 746
Uterus, rupture of 291
Vagina, constricted 696
" inversion of, coming on during labor 745
Valedictory address, by W. E. Horner, M.D., &c., bib. notice of 373
Varicocele, pathology and treatment of 734
Venereal disease, Ricord's illustrations of 131
Ventilation, thermal and other sanitary improvements applicable to public buildings. By Jno. Watson, M.D., &c., bib. notice of 326
Vesico-vaginal fistula 650
Viability, early 676
Vicious advertisements, discouragement of 387
Victim of typhus fever 466
Woman, her diseases and remedies, a series of letters to his class. By Chas. D. Meigs, M.D., &c., bib. notice of 63
Worms, perforation of intestinal canal by 687
Wound of the abdomen 489
" gun-shot, case of 117
" of stomach 162

